# The Influence of Fibrous Elastomer Structure and Porosity on Matrix Organization

**DOI:** 10.1371/journal.pone.0015717

**Published:** 2010-12-22

**Authors:** Jamie L. Ifkovits, Katherine Wu, Robert L. Mauck, Jason A. Burdick

**Affiliations:** 1 Department of Bioengineering, University of Pennsylvania, Philadelphia, Pennsylvania, United States of America; 2 McKay Orthopaedic Research Laboratory, Department of Orthopaedic Surgery, University of Pennsylvania, Philadelphia, Pennsylvania, United States of America; Université de Technologie de Compiègne, France

## Abstract

Fibrous scaffolds are finding wide use in the field of tissue engineering, as they can be designed to mimic many native tissue properties and structures (e.g., cardiac tissue, meniscus). The influence of fiber alignment and scaffold architecture on cellular interactions and matrix organization was the focus of this study. Three scaffolds were fabricated from the photocrosslinkable elastomer poly(glycerol sebacate) (PGS), with changes in fiber alignment (non-aligned (NA) versus aligned (AL)) and the introduction of a PEO sacrificial polymer population to the AL scaffold (composite (CO)). PEO removal led to an increase in scaffold porosity and maintenance of scaffold anisotropy, as evident through visualization, mechanical testing, and mass loss studies. Hydrated scaffolds possessed moduli that ranged between ∼3–240 kPa, failing within the range of properties (<300 kPa) appropriate for soft tissue engineering. CO scaffolds were completely degraded as early as 16 days, whereas NA and AL scaffolds had ∼90% mass loss after 21 days when monitored *in vitro*. Neonatal cardiomyocytes, used as a representative cell type, that were seeded onto the scaffolds maintained their viability and aligned along the surface of the AL and CO fibers. When implanted subcutaneously in rats, a model that is commonly used to investigate in vivo tissue responses to biomaterials, CO scaffolds were completely integrated at 2 weeks, whereas ∼13% and ∼16% of the NA and AL scaffolds, respectively remained acellular. However, all scaffolds were completely populated with cells at 4 weeks post-implantation. Polarized light microscopy was used to evaluate the collagen elaboration and orientation within the scaffold. An increase in the amount of collagen was observed for CO scaffolds and enhanced alignment of the nascent collagen was observed for AL and CO scaffolds compared to NA scaffolds. Thus, these results indicate that the scaffold architecture and porosity are important considerations in controlling tissue formation.

## Introduction

Scaffolds, along with cells and soluble factors, are key components for the production of a successful tissue engineered construct. Specifically, scaffolds serve as templates to guide nascent matrix formation both *in vitro* and *in vivo* [1]. In addition to being non-toxic, non-immunogenic, and biocompatible, the mechanics and degradation of a scaffold should be easily tuned as desired for a particular application. Furthermore, the size and architecture of the scaffold should be considered such that the diffusion of nutrients and waste can take place and vascular network formation is possible if deemed necessary [2], [3]. The lack of scaffolds that meet all of these criteria for soft tissue engineering (e.g., modulus <300 kPa) has prompted the development of alternative materials and scaffold fabrication techniques.

With this in mind, we recently modified the synthesis of the biodegradable elastomer poly(glycerol sebacate) (PGS) to introduce the reactive acrylate group (Acr-PGS) and therefore capitalize on photopolymerization, making complex scaffold fabrication using electrospinning possible [4], [5]. Synthetic elastomeric scaffolds may be useful for a range of tissues due to their ability to support mechanical deformations while maintaining structural integrity, similar to native elastic tissues, such as blood vessels and cardiac muscle [6]. With this polymer system, dramatic differences in the mechanics (∼60 kPa–1 MPa) and degradation kinetics (20–100% mass loss at 12 weeks) of both bulk polymers and electrospun scaffolds can be obtained with photopolymerized PGS materials through simple changes in molecular weight and % acrylation during synthesis [4], [5]. Thus, it is relatively easy to modulate the mechanical properties of an Acr-PGS based scaffold in order to alleviate any compliance mismatch problems that may exist at an implant site. This is not possible with the use of other synthetic polymers which may be stiffer (e.g., poly(caprolactone)) or less mechanically robust (e.g., poly(ethylene glycol)) than dynamic tissues such as cardiac muscle [7], [8].

Electrospinning is an attractive scaffold processing technique for tissue engineering applications since it is a relatively simple process and because the formed fibers can be aligned to mimic the anisotropic nature of fiber aligned tissues, such as the meniscus of the knee, the annulus fibrosus of the intervertebral disc, and cardiac muscle [1], [9], [10]. Moreover, the electrospinning process is also compatible with photopolymerizable materials [4], [11], [12], [13], with the introduction of an initiator to the electrospinning solution and then light after fiber formation. Fibers are aligned in the electrospinning process by collection onto a rotating mandrel [14], [15], [16], [17], [18]. Alignment of the fibers has been shown to influence cellular interactions by providing structural cues to facilitate orientation of cells along the fiber [15], [19], [20]. Furthermore, cells cultured on aligned scaffolds have been shown to produce organized matrix *in vitro*, and thus improve the mechanical properties of the various constructs for their intended application [15], [21]. However, the influence of fiber alignment on matrix elaboration *in vivo* remains to be fully explored.

One limitation in aligned, electrospun scaffolds is the reduction in porosity of the scaffold due to the dense packing of fibers, which can decrease cellular infiltration throughout the depth of the scaffolds. Investigators have attempted to improve the porosity of the scaffolds for better cellular infiltration using several means, including using a rotating frame collector for fibers [22], layered hydrospinning, [23] and electrospinning of salt particles into scaffolds, which are subsequently leached from the system [24], [25]. An improvement in cellular population and remodeling has also been observed *in vivo* upon inclusion of a more degradable synthetic component (e.g., glycolide) [26] or naturally derived urinary bladder matrix [27] in the polymer blend. In another approach, Baker and colleagues [14] simultaneously electrospun poly(caprolactone) (PCL) and the water soluble poly(ethylene oxide) (PEO) from two separate jets to create a composite scaffold that showed an increase in porosity when the sacrificial PEO fibers were leached from the system with incubation in aqueous medium. A significant increase in cell infiltration for samples that consisted of greater than 40% of PEO by mass was observed *in vitro*. However, greater than 60% PEO by mass compromised the structural integrity of the scaffold [14].

The goal of this work was to further develop and characterize Acr-PGS scaffolds for soft tissue engineering applications, such as a cardiac patches for cellular delivery. Specifically, the impact of scaffold structure (via fiber alignment) and porosity (via introduction of a sacrificial fiber population) on cellular interactions and alignment was evaluated *in vitro* using neonatal cardiomyocytes as a model cell type that is aligned natively in cardiac muscle. Furthermore, the influence of structure and porosity on cellular infiltration as well as matrix elaboration and organization was evaluated using a subcutaneous implant as a model to study tissue organization *in vivo*. This work serves as an initial step towards the development of Acr-PGS scaffolds for the engineering of fiber aligned soft tissues, such as cardiac muscle, the meniscus of the knee, and the annulus fibrosus of the intervertebral disc.

## Results

### Macromer Synthesis and Scaffold Fabrication

**Figure 1:**
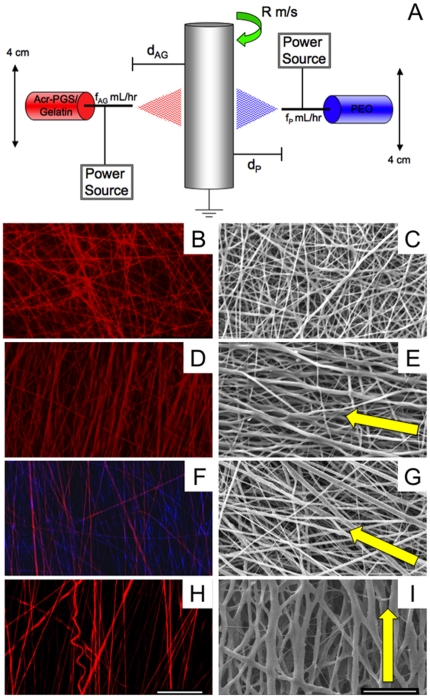
Composite scaffold fabrication schematic and representative electrospun scaffold images. Schematic of dual polymer electrospinning set up (A). Representative fluorescent (Acr-PGS  =  red, PEO  =  blue) and SEM images of NA (B, C), AL (D, E), and CO (F,G) scaffolds following crosslinking and CO scaffolds following PEO removal and lyophilization (H, I). Scale bar = 100 µm (B, D, F, and H) or 20 µm (C, E, G, I).

Acr-PGS was synthesized by a condensation reaction of glycerol and sebacic acid followed by reaction with acryloyl chloride in the presence of triethylamine. A macromer with a M_W_ of 26 kDa and 12.7% acrylation was synthesized, which resulted in a bulk polymer with a modulus of 253.9±18.6 kPa when evaluated using uni-axial tensile testing. The impact of scaffold architecture and porosity on cellular interactions was evaluated using three Acr-PGS scaffolds containing comparable masses of Acr-PGS and the carrier polymer gelatin. One scaffold with non-aligned, randomly oriented fibers (NA) was fabricated by electrospinning an Acr-PGS/gelatin solution onto a flat collection plate. A second Acr-PGS/gelatin scaffold consisting of aligned fibers (AL) was fabricated by collecting electrospun fibers onto a rotating mandrel. Finally, a third scaffold consisting of a mixture of aligned distinct Acr-PGS/gelatin fibers and PEO fibers (CO) was fabricated using the device shown in Figure 1A. Representative fluorescent and SEM images of each scaffold are shown in Figure 1B–I, with the arrow indicating the direction of fiber alignment for the aligned scaffolds (Figure 1E, G, I). An increase in scaffold porosity was observed following PEO removal (Figure 1H–I). Furthermore, an increase in fiber diameter for AL (1.2±0.4 to 1.8±0.6) and CO (1.2±0.4 to 1.7±0.6) scaffolds was also observed following PEO removal treatment and lyophilization.

In order to verify the mass composition of the composite scaffolds, all three scaffolds were subjected to the wash protocol by incubation in DI water for 3 hours and PBS overnight. NA and AL scaffolds lost 17.5±2.2 and 12.3±2.3% mass, respectively, due to loss of unreacted macromer and gelatin. However, CO scaffolds lost 63.0±0.5% mass during processing for PEO removal. Based on the mass loss difference between the AL and CO scaffolds the composition of the CO scaffold was confirmed to be ∼50% Acr-PGS/gelatin and ∼50% PEO.

### Mechanical Properties and Degradation

**Figure 2:**
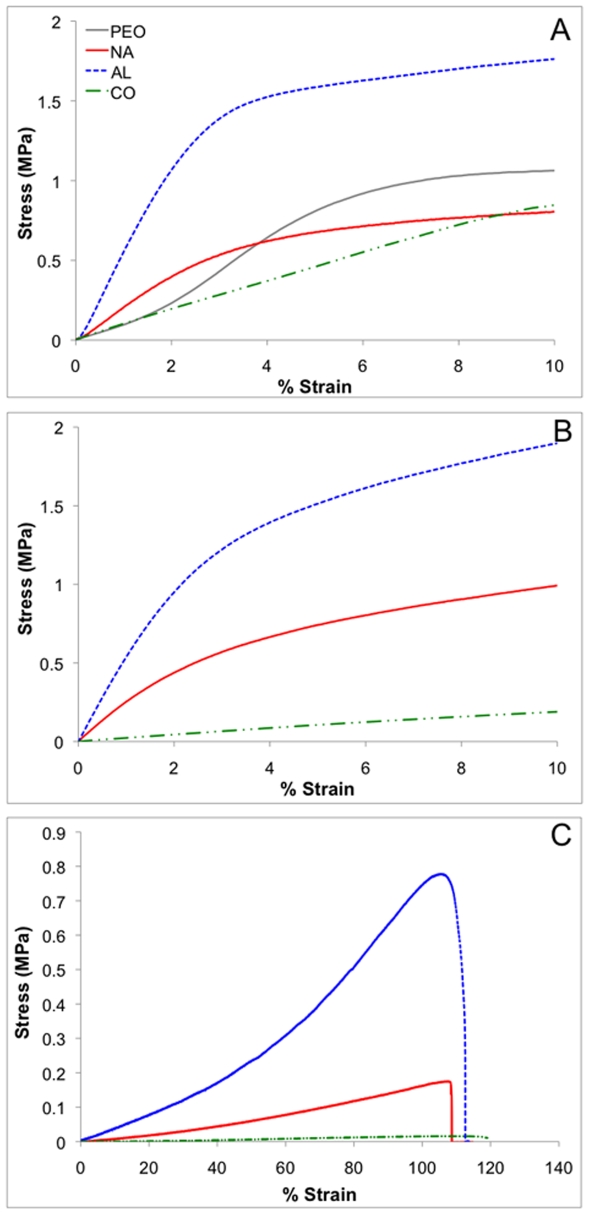
Representative stress versus strain curves. Profiles for scaffolds following photocrosslinking up to 10% strain (A), lyophilization after PEO removal up to 10% strain (B), and hydration in PBS following PEO removal up to failure (C). All profiles are for samples tested parallel to fiber direction.

Uniaxial tensile testing was conducted on all of the scaffolds both in the fiber direction (PA) and transverse to the fiber direction (PE). The anisotropy ratio for the AL and CO scaffolds was calculated as the ratio of the PA modulus to the PE modulus (Table 1). Representative PA stress versus strain curves of scaffolds tested following crosslinking, lyophilization after PEO removal in the dry state, and following PEO removal and incubation in PBS (the hydrated state) are shown in Figure 2.

**Table 1:** Anisotropy ratio of AL and CO scaffolds.

Scaffold	Post-Crosslinking	Post-Wash, Dry	Post-Wash, Hydrated
AL	3.2±0.2	1.7±0.3	2.5±0.3
CO	3.4±0.2	3.1±0.4	6.9±0.4

**Figure 3:**
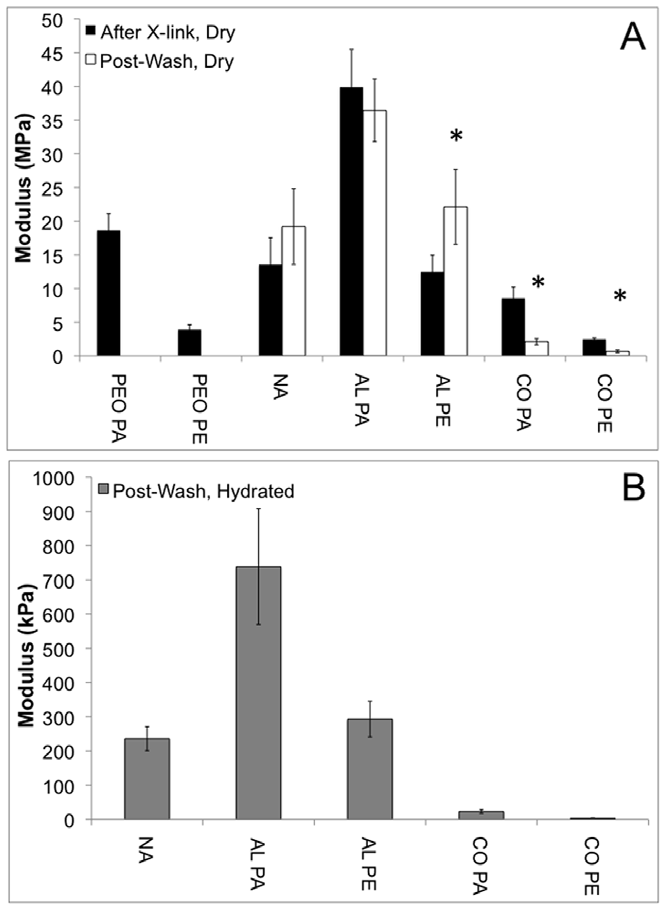
Young's moduli for PEO, NA, AL, and CO scaffolds. Scaffolds were tested in parallel (PA) and perpendicular (PE) fiber directions after photocrosslinking (A, black), after PEO removal and lyophilization (A, white), and after hydration in PBS following PEO removal (B, grey). * denotes p<0.05 for post-wash dry scaffolds (white) versus after crosslinking (black). All scaffolds were significantly different for hydrated scaffolds compared to post-wash dry scaffolds.

PEO scaffolds were also tested for comparison to the CO scaffolds. The modulus of the CO scaffolds (8.5±1.7 MPa and 2.5±0.2 MPa, PA and PE, respectively) tested after crosslinking was less than both the PEO (18.7±2.4 MPa and 3.9±0.7 MPa, PA and PE, respectively) and AL Acr-PGS scaffolds (39.9±5.6 MPa and 12.5±2.5 MPa, respectively) in both the PA and PE directions (Figure 2, 3A). The modulus of the CO scaffolds was drastically reduced after PEO removal (2.1±0.5 MPa and 681.3±219.7 kPa, PA and PE, respectively, Figure 2, 3A), whereas the modulus of the NA and AL scaffolds did not significantly decrease as expected (Figure 2, 3A).

In general, the moduli of the AL scaffolds tested in the PA direction were greater than the NA scaffolds when tested in all conditions. Also, the moduli of all of the scaffolds were significantly reduced upon incubation in water (Figure 2, 3). Furthermore, the % strain at break was increased to approximately 100% for all scaffolds when tested in the hydrated state, without significant differences between the groups (Figure 2).

**Figure 4:**
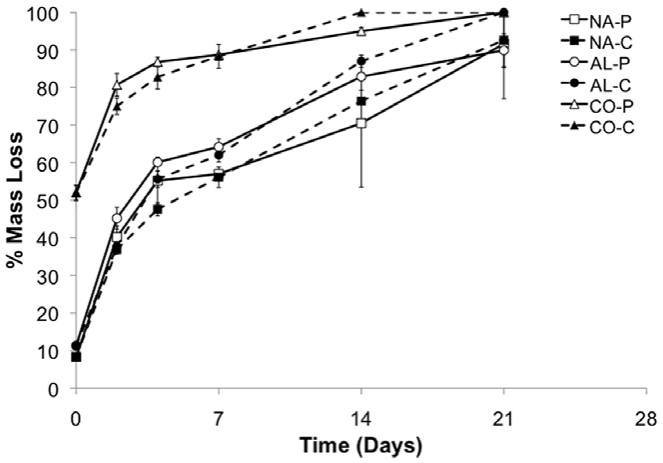
*In vitro* degradation kinetics. Scaffold (NA, AL, and CO) mass loss was monitored after processing for PEO removal (Day 0) in PBS (P, hallow symbols and solid line) or in 0.25 mg mL^−1^ collagenase in PBS (C, filled symbols and dashed line).

The *in vitro* degradation behavior of the different scaffold groups was evaluated following the 18 hour wash period for PEO removal. As expected, NA and AL scaffolds lost ∼10% mass, whereas CO scaffolds lost ∼55% mass after PEO removal (Day 0, Figure 4). The degradation kinetics of scaffolds in PBS as well as PBS containing 0.25 mg mL^−1^ collagenase were evaluated over 21 days. The % mass loss of samples from all scaffold groups was not statistically different upon addition of collagenase to the solution. Furthermore, differences in % mass loss between the NA and AL scaffolds, regardless of the degradation solution, were not observed.

### *In Vitro* Cellular Interactions

**Figure 5:**
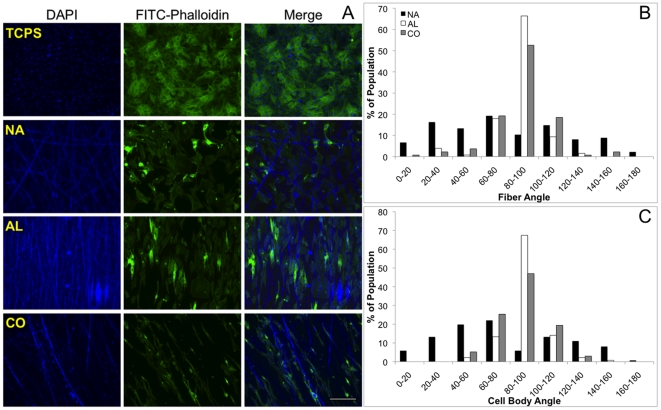
Neonatal cardiomyocyte interactions with TCPS, NA, AL, and CO scaffolds five days after seeding. Cells are stained with FITC-phalloidin for actin fiber visualization and DAPI, which stains both nuclei and Acr-PGS/gelatin fibers (A). Histograms depicting fiber (B) and cellular (C) alignment. Scale bar = 100 µm.

Neonatal cardiomyocytes were seeded onto thin (<100 µm) scaffolds, which were electrospun onto glass coverslips to confirm the ability of fiber orientation to influence cellular alignment with Acr-PGS/gelatin scaffolds. As expected from previous studies with Acr-PGS/gelatin fibrous scaffolds [4], the cells maintained viability over the course of the 5-day study and appeared to interact with the fibers, which is visualized with actin staining (Figure 5A). However, a decrease in cell density was observed for CO scaffolds (82.9±27.6 cells for a given field) compared to NA (135.1±23.3 cells for a given field) and AL (162.2±25.2 cells for a given field) scaffolds. The alignments of the fibers and cells were determined by drawing a horizontal reference line across the images and determining the angles at which a given fiber or cell were viewed with respect to the reference line (Figure 5B). Similar trends were observed upon calculation of the fiber and cellular alignment on the scaffolds, where the AL and CO scaffolds supported highly aligned fibers and cells, whereas the NA scaffold did not (Figure 5B–C).

### *In Vivo* Cellular Interactions

**Figure 6:**
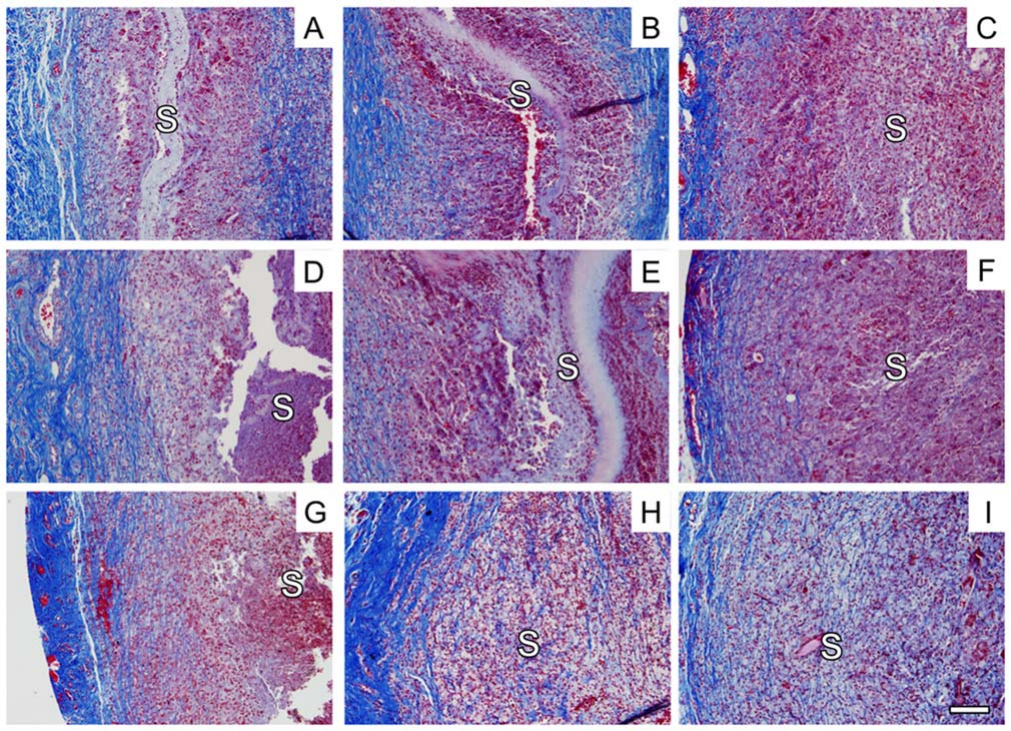
Trichrome stained images of subcutaneous implant samples at 2, 3, and 4 weeks post-implantation. Images were collected for NA (A, D, G), AL (B, E, H), and CO (C, F, I) samples at 2 (A–C), 3 (D–F), and 4 (G–I) weeks post implantation to evaluate integration. S denotes the scaffold region. Scale bar = 50 µm.

Electrospun scaffolds were processed for PEO removal and sterilization prior to implantation into dorsal subcutaneous pockets in rats. Samples were collected at 2, 3, and 4 weeks after implantation and processed using standard histological techniques. Complete integration of the CO scaffolds was observed as early as 2 weeks following implantation (Figure 6C). However, 13.4±7.4% and 16.3±10.8% of free (acellular) scaffold remained for NA and AL scaffolds at 2 weeks, respectively (Figure 6A, B). Similar observations were made when scaffolds were observed at three weeks post-implantation (Figure 6D–F), where 2.1±3.6% and 3.3±5.7% of free scaffold remained for NA and AL scaffolds, respectively (Figure 6.6D, E). Complete integration was observed at 4 weeks for all groups.

**Figure 7:**
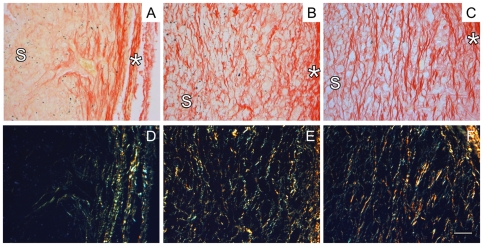
Picrosirius red and polarized light stained images at 4 weeks post-implantation. Images were collected for NA (A, D), AL (B, E), and CO (C, F) samples at 4 weeks post-implantation as viewed using brightfield (A–C) and polarized light microscopy (D–F). S denotes the scaffold region that was used for quantification and * denotes the edge of the scaffold. Scale bar = 50 µm.

**Figure 8:**
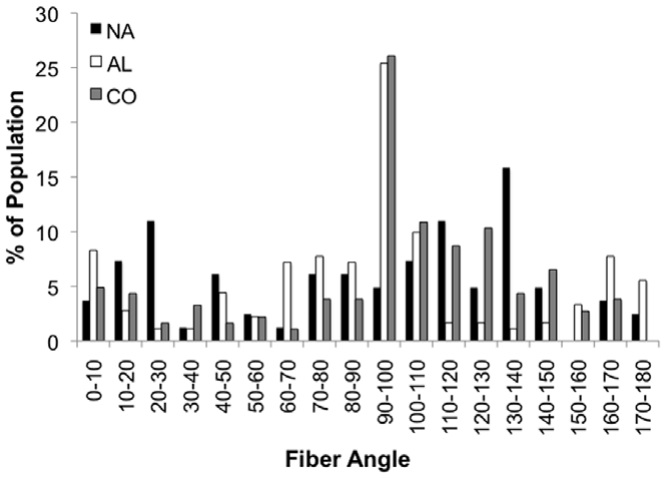
Quantification of collagen fiber alignment. Histogram representing the quantification of collagen fiber alignment within the NA (black), AL (white), and CO (grey) scaffold regions at 4 weeks post-implantation.

In order to evaluate the organization of matrix that developed within the scaffold following subcutaneous implantation, samples were collected 4 weeks post-implantation and sectioned along the fiber direction. A greater quantity of collagen was evident in CO scaffolds (Figure 7C, F) compared to NA and AL scaffolds (Figure 7A, D and Figure 7B, E, respectively). The alignment of collagen fibers located within the scaffolds was quantified for all scaffolds (Figure 8). As expected, the collagen that was elaborated within the AL and CO scaffolds appears to be oriented in an aligned fashion, whereas the collagen found within the NA scaffolds is not (Figure 8).

## Discussion

Within the field of tissue engineering, scaffolds play a key role in guiding the growth and organization of nascent tissue formation. Similar to the native extracellular matrix, scaffolds should be able to facilitate the necessary crosstalk that occurs with the surrounding cells. Electrospinning is a simple, cost-effective technique to create fibrous scaffolds with a similar size scale and architecture to the native extracellular matrix, which has been shown to influence matrix elaboration and organization *in vitro* [1], [15], [21]. Moreover, formation of composite electrospun scaffolds consisting of a stable fiber population and a sacrificial fiber population, which is subsequently dissolved following fabrication, have been used to improve cellular infiltration, a common problem for electrospun scaffolds consisting of aligned fibers [14]. The specific focus of this study was to evaluate the impact of fiber orientation and scaffold porosity on cellular population and matrix organization both *in vitro* and *in vivo* as a subcutaneous implant as a first step towards understand tissue formation and organization *in vivo*. Our eventual goal is to utilize the results from this work to further develop fibrous scaffolds with better understanding of the influence of scaffold structure, material mechanics, and degradation for use in regeneration of fiber aligned soft tissues, such as the myocardium.

The biodegradable, photocrosslinkable Acr-PGS was synthesized via a condensation reaction of glycerol and sebacic acid, followed by reaction with acryloyl chloride. The network properties (i.e., mechanics and degradation kinetics) of the resulting Acr-PGS bulk polymers and fibrous scaffolds can be easily adjusted through simple alterations in molecular weight and % acrylation during synthesis [4], [5]. Although the tunable nature of Acr-PGS makes it attractive for a variety of tissue engineering applications, only one formulation was used in these studies, which was intended to produce scaffolds with moduli suitable for the engineering of soft tissues (i.e., 50–300 kPa) [4], [28].

The influence of scaffold architecture and porosity on cellular interactions and matrix formation was evaluated using three electrospun scaffolds that were fabricated with the same mass of Acr-PGS and the carrier polymer gelatin, but variations in fiber alignment (NA versus AL) and scaffold porosity (CO). As described in previous work, a carrier polymer, gelatin, is needed to facilitate fiber formation with the relatively low molecular weight reactive Acr-PGS during electrospinning [4]. Also, based on the study by Baker *et al*. [15], the composite scaffold was designed to be 50% Acr-PGS/gelatin and 50% PEO by mass, in order to increase the porosity of the scaffold, and thus cellular infiltration without sacrificing the mechanical integrity of the scaffold. The presence and distribution of the two distinct fiber populations was verified using fluorescence microscopy (Figure 1F). The increase in porosity obtained after removal of the sacrificial PEO fibers via washing was also confirmed using fluorescence as well as scanning electron microscopy (Figure 1H–I). Furthermore, the composition of the scaffolds was verified to be ∼50% Acr-PGS/gelatin and ∼50% PEO through mass loss evaluation before and after PEO removal. An increase in fiber diameter was also evident after PEO removal treatment for both AL and CO scaffolds. This is most likely due to water adsorption by the gelatin component causing some swelling of the fibers. As previously described [4], the Acr-PGS/gelatin fibers represent an interpenetrating network of the Acr-PGS intertwined with the gelatin carrier polymer. Although it is likely that there is some loss of gelatin and unreacted Acr-PGS during the wash procedure to remove PEO, some gelatin remains within the network and is capable of adsorbing water.

PEO removal was also confirmed by monitoring the mechanical properties of the scaffolds using uni-axial tensile testing after photocrosslinking and after PEO removal and lyophilization in the dry state. AL and CO scaffolds were tested both parallel to (PA) and perpendicular to (PE) the fiber direction in order to evaluate the anisotropic nature of the scaffolds, with representative stress vs. strain curves for scaffolds tested in the PA direction shown in Figure 2. As expected, the modulus of the CO scaffolds was significantly reduced after PEO removal, whereas the modulus of NA and AL scaffolds was not (Figure 2A–B, Figure 3A). Surprisingly, the modulus of the CO scaffolds after crosslinking was less than both the AL Acr-PGS/gelatin and aligned PEO scaffolds in both directions (Figure 2, Figure 3A). During photopolymerization, the majority of the crosslinking occurs within a given fiber (intra-fiber crosslinking). However, inter-fiber crosslinking, particularly at fiber-fiber junctions, also most likely occurs. The reduction in the CO modulus relative to the AL modulus may be due a reduction in inter-fiber crosslinking that occurs as a result of PEO incorporation. Also unexpectedly, the modulus of the NA scaffold is similar to that of the AL PE, as opposed to between that of AL PA and PE. This may be due an increase in the fiber density of the AL scaffolds, which results in an increase in inter-fiber crosslinking compared to the NA scaffolds.

Prior work demonstrated a dependence of mechanics on hydration state [4], which was also observed in this study. Since the scaffolds have been designed for application in the hydrated environment that exits *in vivo*, it is essential to also understand their mechanical properties in this state. Scaffolds tested after PEO removal and incubation in PBS for one hour demonstrated a significant reduction in the modulus in both the PA and PE directions compared to when tested after PEO removal and lyophilization (Figure 2, Figure 3B). Furthermore, an increase in the % strain at break was observed, without significant differences between the scaffold groups. Although the gelatin component only constitutes ∼23% by mass of the Acr-PGS/gelatin blend, previous work indicates that gelatin dominates the mechanics in the dry state, whereas the Acr-PGS dominates the mechanics in the hydrated state [4]. This is most likely due to water adsorption by the gelatin component of the interpenetrating network that exists and forms a resilient composite upon incubation in aqueous medium. The dramatic increase in the anisotropic ratio of the CO scaffolds in the hydrated state (Table 1) may also be due to an increase the flexibility of the fibers as a result of water adsorption by the gelatin component.

The degradation kinetics of the scaffolds *in vitro* was also evaluated. As expected, an initial increase in mass loss was observed for CO scaffolds at Day 0, due to the loss of the PEO component of the scaffold (Figure 4). The initial mass loss at Day 0 for the NA and AL scaffolds is most likely due to loss of some gelatin and unreacted macromer. CO scaffolds were observed to be completely degraded at Day 16, whereas NA and AL scaffolds had ∼90% mass loss at Day 21 (Figure 4). In order to evaluate potential changes in mass loss that may occur *in vivo* due to the present of enzymes, the degradation kinetics were also evaluated in the presence of 0.25 mg mL^−1^ collagenase. However, no significant differences in mass loss with addition of collagenase were observed. Moreover, significant differences between the mass loss of AL and NA scaffolds, regardless of solution, were not observed over the course of the study. The lack of significant differences in the degradation kinetics with and without collagenase treatment may be due to the fact that all of the scaffold samples were subjected to the same PEO removal protocol. Therefore, it is likely that some of the gelatin diffuses from the scaffolds during this period. Furthermore, any remaining gelatin is likely entrapped within the interpenetrating network that exists with the gelatin and Acr-PGS, making diffusion of the collagenase or any resulting degradation by-products difficult. An advantage to this is that the fibrous scaffold properties can be tailored based on the Acr-PGS chemistry and structure, rather than the gelatin component.

The ability of the scaffold architecture to influence cellular alignment was confirmed *in vitro* using neonatal cardiomyocytes as an example of a cell that is aligned in its native state in the myocardium. Surprisingly, there was a reduction in the cell density on the CO scaffolds compared to the NA and AL scaffolds. However, previous work [4] demonstrated a lack of significant difference in cellular viability of human mesenchymal stem cells seeded on similar Acr-PGS scaffolds compared to control samples. Therefore, the reduction in cell attachment compared for the CO samples compared to the NA and AL scaffold samples may be due to preferential binding to the fibers, possibly due to any residual PEO that may have remained on the glass after washing, and the limited fiber density available for adhesion. The alignment of the fibers and cells were evaluated at 5 days post-seeding by determining the angle of intersection of a fiber or cell body with a horizontal reference line. As expected, a Gaussian distribution of fiber angles was observed for the AL and CO scaffolds, with the greatest quantity of fibers being oriented perpendicular to the reference line. However, this was not the case for the NA scaffolds, where as expected, the fibers displayed a random orientation (Figure 5B). Similar trends in cellular alignment were also observed. The majority of the cells seeded on the AL and CO scaffolds were perpendicular to the reference line, where the cells on the NA scaffold maintained a random orientation (Figure 5C). Thus, Acr-PGS scaffolds have demonstrated the ability to influence cellular organization based on scaffold structure. This, along with the tunable mechanical properties of Acr-PGS scaffolds makes them attractive for use towards a variety of *in vitro* (e.g., improvement in cardiomyocyte electrical conduction) and *in vivo* (e.g., cardiac grafts) applications.

Upon *in vivo* implantation into the subcutaneous dorsal pocket of a rat model, complete integration of the CO scaffolds was observed at 2 weeks post-implantation (Figure 6C), whereas the NA and AL scaffolds remained partially acellular until the 4 week time point (Figure 6). We have previously shown that scaffold thickness can impact *in vivo* degradation and cellular infiltration [4]. The increase in cell infiltration and matrix deposition observed earlier in the CO scaffolds is likely due to a combination of increased porosity and scaffold degradation. We suspect that use of a slower degrading material (e.g., a more modified version of Acr-PGS or poly(ε-caprolactone)) may make the impact of scaffold porosity on cellular infiltration even more pronounced.

Finally, the subcutaneous implant was also used as a model to evaluate the ability of the scaffolds to serve as a template to guide tissue organization; specifically the ability to influence the orientation of the nascent elaborated matrix was evaluated using picrosirius red staining and quantitative polarized light microscopy (Figure 7). Scaffolds were evaluated at 4 weeks post-implantation in order to obtain enough mature collagen for quantification. Furthermore, no polymer and complete cellularity of the scaffold region was observed for all of the scaffold groups. In general, an increase in the amount of collagen within the CO scaffolds was observed due to the combination of increased cellular infiltration and material degradation. Although NA scaffolds appeared to have less collagen present than the AL and CO scaffolds, the collagen that is present appears to be non-aligned (Figure 8). As expected, the collagen that was elaborated within the AL and CO scaffolds appears to be oriented in an aligned fashion (Figure 8).

Thus, the major finding for this work is that although the scaffold material has degraded, it successfully served as a template to direct the organization of the nascent collagen from the infiltrating cells *in vivo*. This demonstration of the ability of a scaffold's architecture to guide matrix formation and orientation *in vivo* is essential in many traditional tissue engineering strategies. For instance, the use of implanted cells after myocardial infarction has only been minimally successful *in vivo*. This may be due to the lack of structural cues necessary to induce the proper cellular alignment due to the disorganized scar that develops after infarct [29]. Although it is well understood that the environment that exists subcutaneously is different than that of different regions of interest for the engineering of soft tissues, such as the myocardium [30], we would expect to also observe similar alignment of the nascent collagen with use of the AL or CO scaffolds at a different site *in vivo*.

This study focused on the manipulation of electrospun scaffold architecture (via control of fiber alignment) and porosity (via inclusion of a sacrificial fiber population) to control cellular interactions *in vitro* as well as cellular population and matrix organization *in vivo*. Our findings clearly demonstrate that scaffold porosity can be increased upon inclusion of a sacrificial fiber population and that mechanical anisotropy and control over cellular interactions (i.e., alignment) can still be maintained upon removal of the sacrificial fiber population. The manipulation of scaffold porosity led to an improvement in cellular population and matrix elaboration *in vivo*. Moreover, manipulation of the scaffold architecture can be used to control the organization of the nascent collagen that is elaborated *in vivo.* The tunable mechanical properties, degradation kinetics, and now scaffold architectures possible with Acr-PGS/gelatin scaffolds make them attractive candidates for a variety of soft tissue engineering applications, particularly for fiber aligned tissues such as the myocardium, meniscus, and annulus fibrosus.

## Materials and Methods

### Ethics Statement

All animal work was conducted under a protocol (802647) approved by the University of Pennsylvania Institutional Animal Care and Use Committee.

### Acr-PGS Macromer Synthesis

All reagents were purchased from Sigma Chemical Company (St. Louis, MO) and used as received unless noted. Acr-PGS was synthesized as previously described [5]. Briefly, the PGS prepolymer was formed by the condensation reaction of equimolar amounts of glycerol (ThermoFisher Scientific, Waltham, MA) and sebacic acid. The reagents were combined at 120°C under nitrogen for 2 hours before a vacuum was applied for 47 hours. For acrylation, the condensation product was dissolved in methylene chloride (1∶10, ThermoFisher Scientific) containing triethylamine (TEA, equimolar to acryloyl chloride) and 500 ppm 4-methoxyphenol (MeHQ) and 15% acryloyl chloride (1∶10 v/v in methylene chloride), which was slowly dripped into the solution. This molar ratio was calculated using the estimation that two of the three hydroxy groups present in glycerol reacted with the sebacic acid during the condensation reaction. An additional 500 ppm MeHQ was added to the reaction flask and a rotary evaporator (40°C, 450 mbar) was used to remove the methylene chloride. Ethyl acetate was added to the reaction flask and the solution was filtered to remove the TEA salts and washed three times with 10 mM hydrochloric acid to remove any remaining salts and unreacted acrylic acid. Ethyl acetate was removed via rotovapping (40°C, 99 mbar) to leave a viscous liquid, which was redissolved in methylene chloride and stored at 4°C. The condensation product molecular weights were verified using GPC (Waters GPC System, Milford, MA) and the acrylation was assessed with ^1^H NMR spectroscopy (Bruker Advance 360 MHz, Bruker, Billerica, MA). Macromers were mixed with 0.5 wt% (with respect to the mass of macromer) of the photoinitiator 2,2-dimethoxy-2-acetophenone (DMPA, 10 wt% in methylene chloride).

### Fabrication of Electrospun Acr-PGS/Gelatin Scaffolds

A solution containing 15 wt% of the macromer/photoinitiator and 4.5 wt% of gelatin B (from bovine skin) in 1,1,1,3,3,3 hexafluoro-2-propanol (HFIP) and a 10 wt% solution of poly(ethylene oxide) (PEO, 200 kDa, Polysciences, Inc. Warrington, PA) in 90% ethanol were prepared for electrospinning. First, a scaffold with non-aligned (NA) fibers was prepared by electrospinning Acr-PGS/gelatin alone at 1.5 mL hr^−1^, +15 kV onto a flat collection plate located 10 cm from the spinnerette. The remaining scaffolds were prepared by electrospinning onto a rotating mandrel (Ø = 2”, 12 m/s) using a custom setup as previously described by Baker *et al*. [14] (Figure 6.1). Briefly, a syringe containing the electrospinning solution was connected to a 5 cm piece of silicone tubing fitted with luer lock attachments and placed in a syringe pump programmed to operate at a flow rate of 1.5 mL hr^−1^. A 6” blunt end 18-gauge needle was then attached to serve as the charged spinnerette. The spinnerette was inserted into a custom-built “fanner,” such that it translated along a 4 cm path across the mandrel. A power supply (Gamma High Voltage, Ormond Beach, FL) was used to apply a potential difference of +15 kV between the spinnerette and the grounded collection apparatus located 10 cm away. The second scaffold was prepared by electrospinning Acr-PGS/gelatin alone (AL) onto the rotating mandrel, such as to generate a scaffold composed of aligned fibers. Next, stable Acr-PGS/gelatin fibers were electrospun concurrently with a second solution containing 10 wt% PEO (200 kDa, Polysciences, Inc. Warrington, PA) in order to introduce a sacrificial fiber population, thus generating a third aligned, composite scaffold (CO). This was accomplished using a second spinneret and fanner located on the opposite side of the mandrel (Figure 6.1). A second power source was connected to this spinnerette and set up to operate using the same conditions described. However, this syringe pump was programmed to operate at 3.0 mL hr^−1^, such that the resulting composite scaffold would contain 50% Acr-PGS/gelatin and 50% PEO by mass. All scaffolds were electrospun for 13 hours. A final scaffold consisting of PEO alone was also electrospun for comparison of mechanical properties. After electrospinning, scaffolds were stored under vacuum overnight. All scaffolds containing Acr-PGS were crosslinked with exposure to ∼10 mW cm^−2^ 365 nm ultraviolet light (Blak-Ray, Ultraviolet Products, Upland, CA) in a nitrogen atmosphere. All scaffolds were also gold sputter coated and viewed using scanning electron microscopy (SEM, Penn Regional Nanotech Facility, JEOL 7500 HR-SEM, Tokyo, Japan).

### Sacrificial Fiber Removal

Scaffolds were washed after fabrication to remove the PEO sacrificial polymer. Samples from the CO group (n = 3 per group) were weighed in the dry state and the PEO fibers were removed by incubation in DI water for three hours, with fresh water changes occurring at each hour, and incubation in phosphate buffered saline (PBS) overnight. The NA and AL scaffolds were used as controls. The dry weight of the scaffolds were then collected following lyophilization (Freezone 4.5, Labconco, Kansas City, MO). SEM images were also taken prior to and following PEO removal. Fibers were also visualized following electrospinning onto glass coverslips for 5 minutes with the fluorescent molecules methacryloxyethyl thiocarbamoyl rhodamine B (50 µM, Polysciences) or DAPI (20 µM) loaded into the Acr-PGS/gelatin or PEO solutions, respectively. Images were taken on a fluorescent microscope (Olympus BX51, Center Valley, PA) following crosslinking and PEO removal.

### Mechanical Testing

All scaffolds were subject to uniaxial tensile testing in both the parallel (PA) and perpendicular (PE) fiber directions after crosslinking as well as after PEO removal and lyophilization. Due to prior work [4] demonstrating an influence of hydration state on scaffold mechanics, samples were also tested at the conclusion of PEO removal, but before drying. All samples for mechanical testing (n = 3–6 per group and 25×5 mm) were cut and uniaxial tensile testing was performed on an Instron 5848 mechanical tester (Canton, MA) at a strain rate of 0.1% s^−1^ following a preload with 0.50 N at 0.05% strain for 60 seconds, and preconditioning with 10 sinusoidal cycles of 0.5% strain at 0.1 Hz prior to testing to failure. The tensile modulus of the construct was calculated from the linear region of the stress-strain curve (0–3%) and initial sample geometry.

### *In Vitro* Degradation

All scaffolds were subject to *in vitro* degradation studies conducted in both PBS and collagenase in PBS. The mass of each sample (n = 3 per group per time point, 5×5 mm) was recorded prior to any treatment (t = Day−1). All samples were then subject to the PEO removal protocol described above (t = Day 0) at which point each scaffold set was divided into two groups. The mass loss of one group in PBS and the other group in 0.25 mg mL^−1^ collagenase in PBS at 37°C was monitored after 2, 4, 7, 14, and 21 days. All solutions were changed every 2–3 days. At each time point, samples were rinsed in DI water two times prior to lyophilization. The mass of each scaffold was recorded after lyophilization and the % mass loss was calculated.

### Cell Adhesion

To investigate cellular interactions with the Acr-PGS/gelatin fibers, samples were prepared by electrospinning each macromer/gelatin solution onto glass coverslips (22×22 mm, Corning, Lowell, MA) for 20 minutes with the previously mentioned electrospinning parameters. To ensure that the fibers adhered to the glass, the coverslips were treated with poly(3-trimethoxysilyl)propyl methacrylate (TMSMA) as was previously described [31] prior to electrospinning. Briefly, coverslips were plasma treated for 3 minutes (700 mAmp, Plasma Prep II, SPI Supplies, West Chester, PA), coated with TMSMA, baked at 100°C for 30 minutes, baked at 110°C for 10 minutes, washed with DI water, and dried overnight. After electrospinning, samples were maintained under vacuum overnight to ensure complete solvent removal, photocrosslinked as above with ultraviolet light (Blak-Ray) in a nitrogen atmosphere, and incubated in PBS overnight. Samples were placed in non-treated wells of a 6-well plate, sterilized upon exposure to germicidal ultraviolet light for 30 minutes, and seeded with freshly isolated neonatal rat cardiomyocytes (50,000 cells cm^−1^), a kind gift from Dr. Kenneth Margulies' lab [32]. Media (DMEM:M199 4:1, 10% horse serum, 5% fetal bovine serum, 1% penicillin/streptomycin/glutamine, and 1% 1 M HEPES, all from Invitrogen, Carlsbad, CA) was replaced every other day and on day 5 cells were rinsed with PBS 3× and fixed in 4% formalin for 10 minutes. The cells were permeabilized with 0.25% Triton X-100 in PBS for 10 minutes, and nonspecific binding sites were blocked with 3% bovine serum albumin and 0.1% Tween-20 in PBS for 15 minutes. Actin stress fibers were stained using FITC-conjugated phalloidin (0.66 µg mL^−1^ in blocking solution) for 40 minutes at 37°C. Cell nuclei were visualized following DAPI staining (2 µg mL^−1^) for 5 minutes. Samples were rinsed three times with PBS at each step and images were taken on a fluorescent microscope (Olympus BX51, Center Valley, PA) with a digital camera (Olympus DP72). Images were post-processed using NIH ImageJ software to calculate fiber (n = 128–135 per group) and cell (n = 128–135 per group) alignment. Cell density was quantified by counting the total number of cells for three representative images for each sample (n = 9 per group) using the NIH ImageJ software.

### *In Vivo* Evaluation

Animals were cared for according to a protocol approved by the University of Pennsylvania Institute for Animal and Use Committee Electrospun scaffolds were processed for PEO removal and sterilized using germicidal ultraviolet light as previously described. Samples (n = 3–6 samples per group, 10 mm length× 5 mm width, NA 0.25–0.6 mm, AL 0.24–0.4 mm, CO 0.88–1.12 mm thick) were collected at 2, 3, and 4 weeks post-subcutaneous implantation into dorsal pockets of rats, fixed in 4% formalin for 24–36 hours, and processed using standard histological techniques. Following paraffin embedding, samples were processed into 7 µm thick sections in the scaffold cross-section, along the direction of the fibers for AL and CO samples. Samples were stained using Masson's trichrome (substituting hematoxylin Gill No. 2 for Weighret's hematoxylin). The amount of free (acellular) scaffold was calculated by determining the thickness of the acellular region of the scaffold (n = 10 per sample) using ImageJ, divided by the initial thickness of the sample, which was normalized to acellular scaffolds that were also processed and visualized using trichrome staining. Any defects that were evident in the sections and resulting images that occurred as a result of the histological processing were excluded from these calculations.

In order to evaluate matrix elaboration, samples were also stained using picrosirius red. Trichrome stained images were obtained on an upright microscope (Olympus BX51) with a digital camera (Olympus DP72) and post-processed using NIH ImageJ to determine the amount of free (lacking cellular infiltration) scaffold at each time point. Quantitative polarized light microscopy was used to visualize and quantify collagen alignment as described in Nerurkar *et al*. [21]. Briefly, greyscale images were collected at 10×in 10° increments using a green band-pass filter (BP 546 nm) with a crossed analyzer and polarizer coordinately rotated through a 90° span on a Leica DM/LP microscope. The filter was replaced with a λ compensator and images were again collected through the same 90° span. A custom program was used to determine the collagen fiber orientation (n = 25–30 collagen fibers for NA, 55–70 collagen fibers for AL and CO) located within the scaffold region per sample.

### Statistical Analysis

All data is presented as mean ± standard deviation. Comparisons using a Student's t-test assuming unequal variances was used for data in which the variances between groups was not equal, otherwise, single factor ANOVA with Tukey's *post hoc* test was used to determine statistical significance among groups with p<0.05.
